# The Expression Modulation of the Key Enzyme Acc for Highly Efficient 3-Hydroxypropionic Acid Production

**DOI:** 10.3389/fmicb.2022.902848

**Published:** 2022-05-11

**Authors:** Sumeng Wang, Xin Jin, Wei Jiang, Qian Wang, Qingsheng Qi, Quanfeng Liang

**Affiliations:** State Key Laboratory of Microbial Technology, Shandong University, Jinan, China

**Keywords:** 3-hydroxypropionic acid, cell growth, adjusting AccBC-DtsR1 level, balancing malonyl-CoA pathway, productivity

## Abstract

3-Hydroxypropionic acid (3-HP) is a promising high value-added chemical. Acetyl-CoA carboxylase (Acc) is a vital rate-limiting step in 3-HP biosynthesis through the malonyl-CoA pathway. However, Acc toxicity in cells during growth blocks its ability to catalyze acetyl-CoA to malonyl-CoA. The balancing of Acc and malonyl-CoA reductase (MCR) expression is another an unexplored but key process in 3-HP production. To solve these problems, in the present study, we developed a method to mitigate Acc toxicity cell growth through Acc subunits (AccBC and DtsR1) expression adjustment. The results revealed that cell growth and 3-HP production can be accelerated through the adjustment of DtsR1 and AccBC expression. Subsequently, the balancing Acc and MCR expression was also employed for 3-HP production, the engineered strain achieved the highest titer of 6.8 g/L, with a high yield of 0.566 g/g glucose and productivity of 0.13 g/L/h, in shake-flask fermentation through the malonyl-CoA pathway. Likewise, the engineered strain also had the highest productivity (1.03 g/L/h) as well as a high yield (0.246 g/g glucose) and titer (up to 38.13 g/L) in fed-batch fermentation, constituting the most efficient strain for 3-HP production through the malonyl-CoA pathway using a cheap carbon source. This strategy might facilitate the production of other malonyl-CoA-derived chemical compounds in the future.

## Introduction

**Figure 1:**
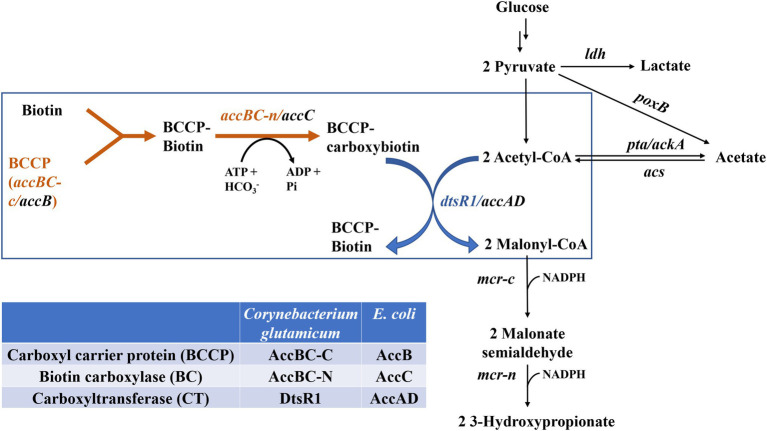
Biosynthesis of 3-HP through the malonyl-CoA pathway by regulating the expression of *Corynebacterium glutamicum*–derived Acc*. ldh*, lactate dehydrogenase; *poxB*, pyruvate oxidase; *pta/ackA*, acetate acetyltransferase/acetate kinase; *acs*, acetyl-coenzyme A synthetase; *mcr-c*, C-terminal of mcr (malonyl-CoA reductase); *mcr-n*, N-terminal of *mcr*.

3-Hydroxypropionic acid (3-HP, CAS 503-66-2) is a precursor for numerous chemicals (such as 1,3-propanediol and malonic acid) and can be polymerized to useful polymers, such as poly(3-HP; Liu et al., 2017). Because 3-HP was listed as one of the top value-added chemicals by the US Department of Energy in 2004 (Werpy et al., 2004), an increasing number of microbiology studies have focused on 3-HP synthesis. Since the early 2000s, various microorganisms, including *Klebsiella pneumoniae* and *Escherichia coli* et al., have been metabolically engineered to produce 3-HP using different substrates (De Fouchécour et al., 2018; Ji et al., 2018; Zhou et al., 2020). Three major routes of 3-HP biosynthesis have been reported, namely the (i) glycerol, (ii) malonyl-CoA, and (iii) β-alanine pathways (Liu et al., 2017; De Fouchécour et al., 2018). Among these, the malonyl-CoA pathway has attracted considerable scientific attention and is particularly advantageous for its redox-neutral properties resulting from the use of a glucose substrate and its thermodynamic feasibility (Liu et al., 2017). In this pathway, 3-HP is synthesized from acetyl-CoA in three steps (Figure 1; De Fouchécour et al., 2018). The first step involves the conversion of acetyl-CoA to malonyl-CoA through acetyl-CoA carboxylase (Acc), and the other two steps consist of two reductions with dissected malonyl-CoA reductase (MCR), including MCR-C and MCR-N, to catalyze malonyl-CoA to 3-HP (Liu et al., 2013; De Fouchécour et al., 2018). MCR derived from *Chloroflexus aurantiacus* was systematically studied through the mutation and balancing of this bifunctional enzyme (Liu et al., 2016). *Escherichia coli*–derived Acc comprises four subunits, two carboxyltransferase (α and β) subunits (encoded by *accA* and *accD*), carboxyl carrier protein (encoded by *accB*), and biotin carboxylase (BC; encoded by *accC*). However, *Corynebacterium glutamicum*–derived Acc consists of only two subunits, AccBC functions as C-terminal carboxyl carrier protein and N-terminal biotin carboxylase, and DtsR1 function as carboxyltransferase (Cheng et al., 2016). One common strategy is to induce Acc overexpression to enhance intracellular malonyl-CoA accumulation for the production of biochemicals such as 3-HP, polyketide triacetic acid lactone, and fatty acid (Davis et al., 2000; Zha et al., 2009; Rathnasingh et al., 2012; Liu et al., 2013, 2016, 2019b; Xu et al., 2014; Han et al., 2020). However, many studies have reported that Acc overexpression is toxic to cells, although the mechanism of this toxicity remains unclear (Davis et al., 2000; Zha et al., 2009; Liu et al., 2015). To alleviate this toxicity, biosensors have been used to adjust Acc expression (Xu et al., 2014; Liu et al., 2015). However, the application of these methods in industrial production is difficult because of genetic circuit instability (Liao et al., 2019). Therefore, investigating more stable new methods is essential for addressing this bottleneck in the biosynthesis of malonyl-CoA derivatives.

This study aimed to mitigate the toxicity of Acc during cell growth and improve 3-HP production. We developed a novel strategy to address the problem of Acc toxicity during cell growth and 3-HP production by modifying *C. glutamicum*–derived DtsR1 and AccBC expression levels and demonstrated the effectiveness of this method. Additionally, balancing the Acc and MCR expression has been used to increase 3-HP production. Finally, the engineered 3-HP-producing *E. coli* strain achieved highest productivity of 1.03 g/L/h and a high yield of 0.246 g/g glucose, with the titer reaching 38.13 g/L during fed-batch fermentation.

## Materials and Methods

### Strains, Plasmids, and Media

All strains and plasmids used in this study are listed in Table 1 and Supplementary Table S1. *Escherichia coli* BL (DE3) was used for exploring the effect of DtsR1 and AccBC expression levels on cell growth, with *E. coli* DH5α used for cloning reconstructed plasmids. Strain Q2098 and strain *E. coli-derived* Acc, a 3-HP producing strain including the overexpressed native *E. coli* Acc, and the plasmid (pMCR-C-N940V/K1106W/S1114R) for 3-HP production were kindly provided by Prof. Guang Zhao. pACYCDuet-1 and pETDuet-1 were purchased from EMD Biosciences (Novagen) for protein expression. Strain with expression of *C. glutamicum*–derived Acc was engineered through the transformation of pA-DtsR1-accBC and pMCR-CN940V/K1106W/S1114R into Q2098. RBS characterization strains, namely BL-0029, BL-0030, BL-0031, BL-0032, BL-0033, BL-0034, BL-0035, BL-0064, and BL-AR, were constructed through the transformation of plasmids pA-0029 carrying RBS B0029, pA-0030 carrying RBS B0030, pA-0031 carrying RBS B0031, pA-0032 carrying RBS B0032, pA-0033 carrying RBS B0033, pA-0034 carrying RBS B0034, pA-0035 carrying RBS B0035, pA-0063 carrying RBS B0064, and pA-AR carrying RBS AR, respectively, into *E. coli* BL (DE3). For cell growth analysis, control, Expressed DtsR1, Expressed AccBC, W-3430, W-34AR, W-3034, W-AR34, W-AR30, W-30AR, W-3030, W-ARAR, and W-3434 strains were constructed through the transformation of plasmids pACYCDuet-1 without Acc expression, pA-DtsR1 carrying *dtsR1*, pA-AccBC carrying *accBC*, D34-A30 carrying B0034-*dtsR1*-B0030-*accBC*, D34-AAR carrying B0034-*dtsR1*-AR-*accBC*, D30-A34 carrying B0030-*dtsR1*-B0034-*accBC*, DAR-A34 carrying AR-*dtsR1*-B0034-*accBC*, DAR-A30 carrying AR-*dtsR1*-B0030-*accBC*, D30-AAR carrying B0030-d*tsR1*-AR-*accBC*, D30-A30 carrying B0030-*dtsR1*-B0030-*accBC*, DAR-AAR carrying AR-*dtsR1*-AR-*accBC* and D34-A34 carrying B0034-*dtsR1*-B0034, respectively, into *E. coli* BL (DE3). To produce 3-HP, the plasmid pMCR-C-N940V/K1106W/S1114R carrying mutated *mcr-c* with *acc* expression plasmid including D34-A30/D34-AAR/D30-A34/DAR-A34/D34-A34/DAR-AAR/D30-A30/D64-A64 were co-transformed into producing strain Q2098 integrated with three copy numbers of *mcr-n* to generate Q-3430/Q-34AR/Q-3034/Q-AR34/Q-3434/Q-ARAR/Q-3030/Q-6464 strains for 3-HP production. Luria–Bertani (LB) medium containing 10 g/L tryptone, 5 g/L yeast extract, and 10 g/L NaCl was used for plasmid construction and cell growth characterization, and modified M9 medium containing 14 g/L K_2_HPO_4_ ▪ 3H_2_O, 5.2 g/L KH_2_PO_4_, 1 g/L NaCl, 1 g/L NH_4_Cl, 0.5 g/L MgSO_4_, 0.2 g/L yeast extract, and 20 g/L glucose was used for single colony cultivation, Acc expression, and shake-flask fermentation. The fed-batch fermentation medium contained 20 g/L glucose, 9.8 g/L K_2_HPO_4_ ▪ 3H_2_O, 3.0 g/L (NH_4_)_2_SO_4_, 2.1 g/L citric acid monohydrate, 0.3 g/L ammonium ferric citrate, 0.5 g/L MgSO_4_, 9 mg/L CaCl_2_ ▪ 2H_2_O, 6 mg/L FeSO_4_ ▪ 7H_2_O, 2 mg/L H_3_BO_3_, 2 mg/L MnCl_2_ ▪ 4H_2_O, 0.8 mg/L (NH_4_)_6_Mo_7_O_24_ ▪ 4H_2_O, and 0.2 mg/L CuSO_4_ ▪ 5H_2_O (Liu et al., 2016).

**Table 1:** Strains used in this study.

Strains	Description	Source
*Strains*		
*Escherichia coli* BL21 (DE3)	F^−^ *ompT gal dcm lon hsdSB* (rB^−^ mB^−^) λ(DE3)	Invitrogen
*E. coli* DH5α	F^−^ supE44 ΔlacU169 (*ϕ80 lacZ ΔM15*) *hsdR*17 *recA*1 *endA*1 *gyrA96 thi*-1 *relA*1	Invitrogen
Q2098	*E. coli* BL21(DE3) Δ*prpR:lacI* P_T7_*His_6_-mcr*_1-549_Δ*melR*:*lacI* P_T7_*His_6_-mcr_1-549_* Δ*mtlA*:*lacI* P_T7_*His_6_-mcr_1-549_*	Liu et al., 2016
*E. coli-derived* Acc	Q2098/pA-accADBC/pMCR-CN940V/K1106W/S1114R	Liu et al., 2016
*Corynebacterium glutamicum*–derived Acc	Q2098 carrying pA-DtsR1-AccBC and pMCR-CN940V/K1106W/S1114R	This study
30AccAD-64AccBC	*E. coli* BL21 (DE3) carrying AD30BC64	This study
34AccAD-30AccBC	*E. coli* BL21 (DE3) carrying AD34BC30	This study
BL-0029	*E. coli* BL21 (DE3) carrying pA-0029	This study
BL-0030	*E. coli* BL21 (DE3) carrying pA-0030	This study
BL-0031	*E. coli* BL21 (DE3) carrying pA-0031	This study
BL-0032	*E. coli* BL21 (DE3) carrying pA-0032	This study
BL-0033	*E. coli* BL21 (DE3) carrying pA-0033	This study
BL-0034	*E. coli* BL21 (DE3) carrying pA-0034	This study
BL-0035	*E. coli* BL21 (DE3) carrying pA-0035	This study
BL-0064	*E. coli* BL21 (DE3) carrying pA-0064	This study
BL-AR	*E. coli* BL21 (DE3) carrying pAAR	This study
Control	*E. coli* BL21 (DE3) carrying pACYCDuet-1	This study
BL-DtsR1	*E. coli* BL21 (DE3) carrying pA-DtsR1	This study
BL-AccBC	*E. coli* BL21 (DE3) carrying pA-AccBC	This study
W-3430	*E. coli* BL21 (DE3) carrying D34-A30	This study
W-34AR	*E. coli* BL21 (DE3) carrying D34-AAR	This study
W-3034	*E. coli* BL21 (DE3) carrying D30-A34	This study
W-AR34	*E. coli* BL21 (DE3) carrying DAR-A34	This study
W-AR30	*E. coli* BL21 (DE3) carrying DAR-A30	This study
W-30AR	*E. coli* BL21 (DE3) carrying D30-AAR	This study
W-3030	*E. coli* BL21 (DE3) carrying D30-A30	This study
W-ARAR	*E. coli* BL21 (DE3) carrying DAR-AAR	This study
W-3434	*E. coli* BL21 (DE3) carrying D34-A34	This study
Q-3430	Q2098 carrying D34-A30 and pMCR-C-N940V/K1106W/S1114R	This study
Q-34AR	Q2098 carrying D34-AR and pMCR-C-N940V/K1106W/S1114R	This study
Q-3034	Q2098 carrying D30-A34 and pMCR-C-N940V/K1106W/S1114R	This study
Q-AR34	Q2098 carrying DAR-A34 and pMCR-C-N940V/K1106W/S1114R	This study
Q-3030	Q2098 carrying D30-A30 and pMCR-C-N940V/K1106W/S1114R	This study
Q-ARAR	Q2098 carrying DAR-AAR and pMCR-C-N940V/K1106W/S1114R	This study
Q-3434	Q2098 carrying D34-A34 and pMCR-C-N940V/K1106W/S1114R	This study
Q-6464	Q2098 carrying D64-A64 and pMCR-C-N940V/K1106W/S1114R	This study

### Plasmid Construction

The primers used for plasmid construction are listed in Supplementary Table S2. All plasmids in this study were constructed using the Gibson assembly method (Gibson et al., 2009). Nine plasmids (pA-0029, pA-0030, pA-0031, pA-0032, pA-0033, pA-0034, pA-0035, pA-0064, and pA-AR) were assembled through the fusion of RFP with RBSs of varying strengths (B0029, B0030, B0031, B0032, B0033, B0034, B0035, and B0064)^1^ and AR from pACYCDuet-1 (Supplementary Table S3) into a pACYCDuet-1 backbone. Two subunits, DtsR1 (1,632 bp and 58.47 KDa, Gene ID: 3345446) and AccBC (1,776 bp and 63.419 KDa, Gene ID: 3343021), of Acc from *C. glutamicum* and *accA* (960 bp and 35.2 KDa, Gene ID: 6062185), *accD* (915 bp and 33.3 KDa, Gene ID: 6059083), *accB* (471 bp and 16.6 KDa, Gene ID: 6058890), and *accC* (1,350 bp and 49.3 KDa, Gene ID: 6058863) from *E. coli* BL21 (DE3) were amplified and assembled into pACYCDuet-1 to generate 13 engineered plasmids to analyze cell growth and 3-HP production. To regulate the expression of DtsR1 and AccBC, plasmids (D30-AAR, D30-A34, DAR-A34, DAR-A30, D34-A30, D34-AAR, D30-A30, DR-AAR, D34-A34, and D64-A64) were constructed using the primers listed in Supplementary Table S2 through the replacement of various RBSs. To balance the expression levels of Acc and MCR for 3-HP production, plasmids containing D34-A30, D34-AAR, D30-A34, DAR-A34, D30-A30, DAR-AAR, D34-A34, and D64-A64 were co-transformed with pMCR-C-N940V/K1106W/S1114R into Q2098. All fragments in this study were amplified with Phanta HS Super-Fidelity DNA Polymerase, which was purchased from Vazyme Biotech (Nanjing, China). All assembled plasmids were transformed into DH5α through the calcium chloride (CaCl_2_) method.

### RBS Characterization and Cell Growth Analysis

For RBS characterization, RFP fluorescence was monitored in real time using a Multi-Detection Microplate Reader (Synergy HT, BioTek, Winooski, VT, United States). Details of the procedure are as follows: The seeds of RBS characterization strains were prepared by transferring single colonies into a 12-well microassay plate with 2 ml of LB medium supplemented with 34 μg/ml chloramphenicol for 12 h at 30°C. Next, 2% of seeds were inoculated into a 96-well microassay plate containing 0.2 ml of LB medium with 34 μg/ml chloramphenicol to detect red fluorescence. The 96-well plate was cultured at 30°C under vigorous shaking. During RBS characterization, the optical density (OD) was measured at 600 nm; red fluorescence was detected through excitation at 590 nm and emission at 645 nm.

For cell growth analysis, single colonies were cultivated in a 12-well microassay plate containing 2 ml of LB medium supplemented with 34 μg/ml chloramphenicol for 12 h at 37°C. Subsequently, 2% seeds were inoculated into a 24-/96-well microassay plate containing 2 ml or 0.2 ml of LB medium with 34 μg/ml chloramphenicol at 30°C to monitor cell growth in real time through detection at an OD of 600 nm. All measurements were performed in triplicate.

### 3-HP Fermentation

Both shake-flask and fed-batch fermentation were performed in different minimal media (Liu et al., 2016). For shake-flask fermentation, a single colony was first cultivated in a 300-mL Erlenmeyer flask with 50 ml of modified M9 medium supplemented with 34 μg/ml chloramphenicol and 100 μg/ml ampicillin sodium at 220 rpm at 37°C for 12 h. Next, 1 ml of preculture was transferred into a 300-mL Erlenmeyer flask containing 50 ml of fresh modified M9 medium with 34 μg/ml chloramphenicol and 100 μg/ml ampicillin sodium at 220 rpm at 37°C. After OD_600_ reached 0.6–0.8, the temperature was controlled at 30°C, and 0.2 mM IPTG, 40 mg/L D-biotin, and 20 mM NaHCO_3_ were added for 3-HP production.

For fed-batch fermentation, the preculture was transferred into fresh modified M9 medium supplemented with 34 μg/ml chloramphenicol and 100 μg/ml ampicillin sodium at 220 rpm at 37°C. Subsequently, 5% (*v*/*v*) of the transferred culture was inoculated into a 7.5-L bioreactor (Infors HT, Bottmingen, Switzerland) containing 4 L of fed-batch fermentation medium. The dissolved oxygen level was controlled at ≥30% through adjustment of the agitation speed and airflow rate. The pH was adjusted to 7.0 by supplying ammonium hydroxide. Glucose was added as carbon source with initial 20 g/L, and supplemented to 20–40 g/L while lower than 10 g/L. Fed-batch fermentation was initially performed at 37°C, and the temperature was downregulated to 30°C when OD_600_ was approximately 15. Simultaneously, 0.2 mM IPTG, 40 mg/L D-biotin, and 20 mM NaHCO_3_ were added for 3-HP production. During fermentation, D-biotin and NaHCO_3_ were supplied every 12 h.

### Analytical Methods

OD was measured at 600 nm with a spectrophotometer (Shimazu, Japan). Samples were centrifuged at 12,000 rpm for 2 min to collect the supernatant. The supernatant was then filtered with a 0.22-μm aqueous membrane for the analysis of 3-HP, glucose, acetate, and lactate. Glucose, acetate, and lactate levels were measured using a high-performance liquid chromatography (HPLC) system (Shimadzu, Kyoto, Japan) equipped with a refractive index detector (RID-10A; Shimadzu, Kyoto, Japan) and an Aminex HPX-87H ion exclusion column (Bio-Rad Laboratories, Hercules, CA, United States); 5 mM H_2_SO_4_ was used as the mobile phase at a flow rate of 0.6 ml/min (Li et al., 2013). The HPLC system equipped with a diode array detector (SPD-M20A; Shimadzu, Kyoto, Japan), with the Aminex HPX-87H ion exclusion column applied at 60°C, was used to determine the 3-HP production level. Finally, 0.5 mM H_2_SO_4_ was used as the mobile phase at a flow rate of 0.4 ml/min. Glucose, acetate, lactate, and 3-HP were identified according to the retention time of standard samples, and the concentration was quantified with the peak area according to the corresponding standard curve.

## Results and Discussion

### Effect of Acc Expression on 3-HP Production

**Figure 2:**
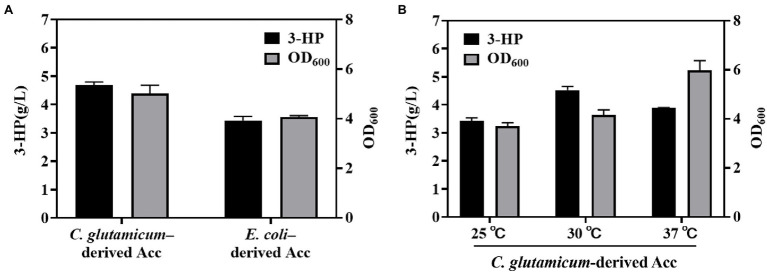
Effect of *Escherichia coli–* and *Corynebacterium glutamicum*–derived Acc and temperature on 3-HP production. **(A)** Comparation of *Corynebacterium glutamicum*–derived Acc and *Escherichia coli*–derived Acc for 3-HP production and cell growth. **(B)** Studying the influence of various temperatures on 3-HP production with strain *Corynebacterium glutamicum*–derived Acc. The fermentation process was performed with a 300-mL shake-flask containing 50 ml of medium (shaken at 220 rpm for 48 h). Cultures were induced with 0.2 mM IPTG when OD_600_ reached 0.6–0.8. All results were calculated with three (*n* = 3) independent replicates.

As a key factor in 3-HP production through the malonyl-CoA pathway, Acc must be suitably screened for the catalyzation of acetyl-CoA to malonyl-CoA. Cheng et al. introduced *C. glutamicum*–derived Acc into *E. coli* BL21 (DE3) and optimized the expression level of Acc and MCR by co-expressing Acc with MCR into the sole plasmid. This resulted in the production of 3-HP up to 1.08 g/L with a yield of 0.18 g/g glucose during flask-shake fermentation (Cheng et al., 2016); however, the yield, productivity, and titer of 3-HP were low. Moreover, the effect of the heterologous expression of AccBC and DtsR1 on cell growth remains unclear. To explore the effect of native and heterologous Acc on 3-HP production, we constructed an *E. coli* BL21 (DE3) strain containing mutated MCR (N940V/K1106W/S1114R; Liu et al., 2016) and Acc from *C. glutamicum*. As depicted in Figure 2A, compared with the strain with expression of native *E. coli*–derived Acc with 3.43 g/L, the engineered strain with expression of heterogenous *C. glutamicum*–derived Acc had a higher 3-HP titer of 4.86 g/L after cultivation at 30°C for 48 h (3-HP retention time at 17.8 min; Supplementary Figure S1). In addition, the cell growth was similar with 3-HP production (Figure 2A and Supplementary Figure S2). This result demonstrated that heterogeneously expressed Acc from *C. glutamicum* is more suitable than *E. coli*–derived Acc for 3-HP production.

Reports have stated that the optimal temperature for mutated MCR-C activity is 72°C (Liu et al., 2016), and the greatest activity of temperature-sensitive AccBC is observable at 30°C (Jäger et al., 1996; Cheng et al., 2016). Thus, we tested the influence of various temperatures (25°C, 30°C, and 37°C) on 3-HP biosynthesis. The highest 3-HP titer was noted at 30°C rather than at 25°C or 37°C. Follow-up experiments were performed at 30°C (Figure 2B).

### Analysis of Cell Growth Through the Adjustment of DtsR1 and AccBC Expression

Studies have indicated that native Acc overexpression in *E. coli* is toxic to cell growth (Davis et al., 2000; Zha et al., 2009; Liu et al., 2015); we verified such growth inhibition using ribosome bind site (RBS) and the inducer isopropyl-β-d-thiogalactoside (IPTG) at various strengths (Supplementary Figure S3).

**Figure 3:**
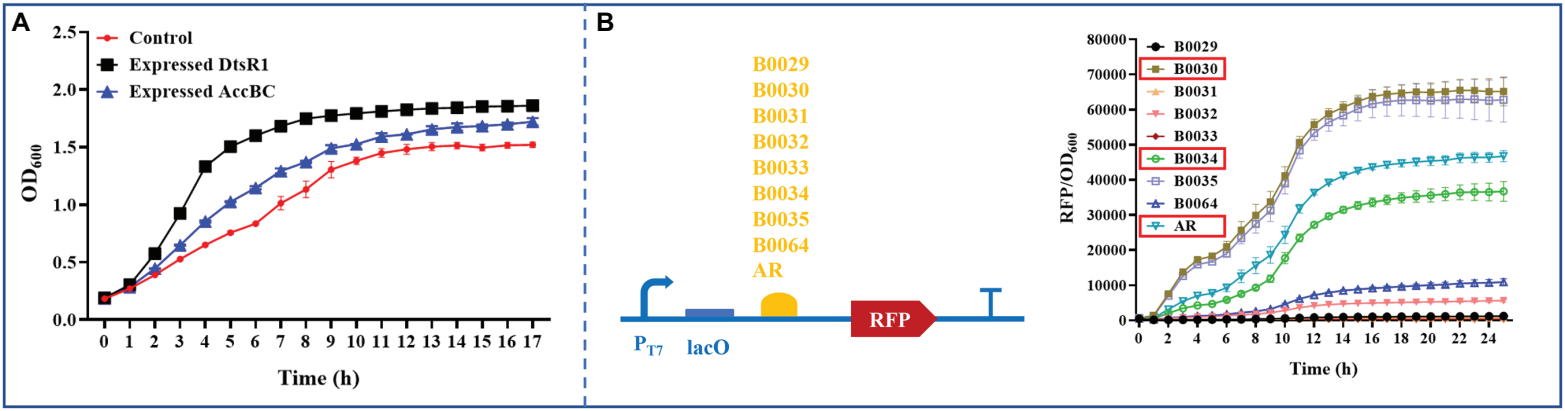
Analyzing the influence of overexpressed DtsR1 or AccBC on cell growth and characterizing RBS strength in *Escherichia coli* BL21 (DE3). **(A)** DtsR1 and AccBC were expressed in strains Expressed DtsR1 and Expressed AccBC, respectively. *Escherichia coli* was cultivated in a 24-well microassay plate containing 2 ml of medium at 30°C; 0.2 mM IPTG was added upon inoculation. Strain containing empty vector as the control. **(B)** RBSs of various strengths were characterized and screened to regulate AccBC and DtsR1 expression through real-time monitoring of the fluorescence intensity of RFP using a Multi-Detection Microplate Reader. RBS strength was calculated with the ratio of RFP to OD_600_. All results were calculated with three (*n* = 3) independent replicates.

**Figure 4:**
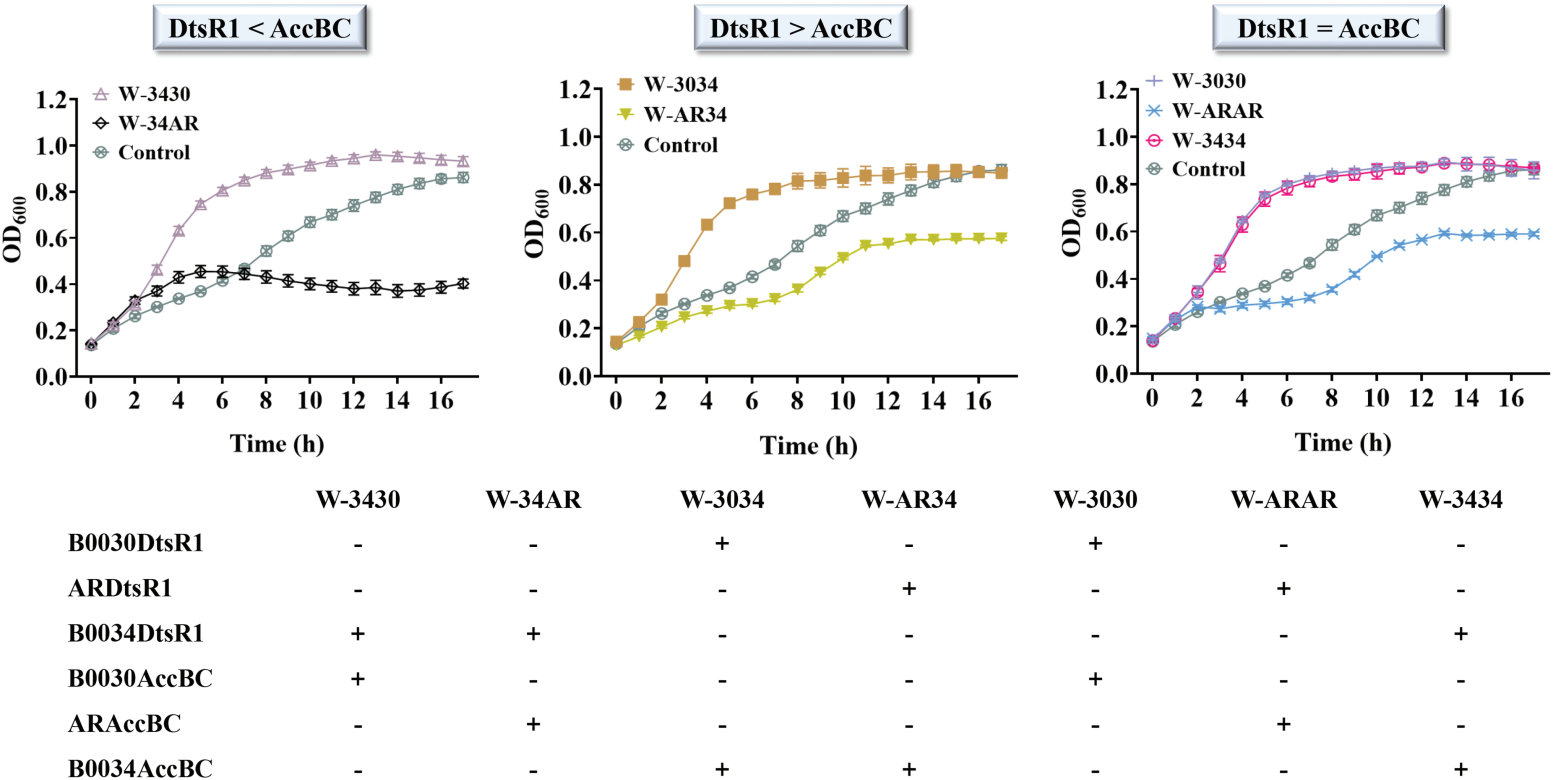
Modified expression of DtsR1 and AccBC and their influence on cell growth in *Escherichia coli* BL21 (DE3). The adjusted DtsR1 and AccBC expression is listed as three levels according to RBS strength: DtsR1 < AccBC, DtsR1 > AccBC, and DtsR1 = AccBC. Strain containing empty vector without Acc expression was the control. The strength of RBS was calculated with the ratio of RFP/OD_600_: 65,116 (B0030), 46,388 (AR), and 36,515 (B0034). All results were calculated with three (*n* = 3) independent replicates.

To improve 3-HP production, we systematically analyzed the influence of overexpressed *C. glutamicum*–derived Acc on cell growth in *E. coli* BL21 (DE3). First, the effect of expressed DtsR1 or AccBC on cell growth was assessed. Compared with the control without expression of DtsR1 or AccBC, both subunits were demonstrably beneficial for cell growth, especially DtsR1 (Figure 3A). To further study the influence of regulated DtsR1 and AccBC on cell growth, the two subunits were expressed in the same plasmid with varying strengths of RBS. Finally, RBSs with different TIR (Translation Initiation Rates), B0030 (65116), AR (46388), and B0034 (36515), respectively, were selected through real-time measurement of the ratio of normalized red fluorescence intensity to cell density (Figure 3B). Accordingly, the strains containing various combinations and levels of AccBC and DtsR1 expression were formed (Figure 4). The results indicated that, compared with the control, different combinations of DtsR1 and AccBC expression levels induce various effects on cell growth. For cells with the expression of AccBC was higher than that of DtsR1, strain W-3430 with high expression level of AccBC grew better than W-34AR with weak expression of AccBC. Similarly, a higher expression level of DtsR1 than that of AccBC lead to increased strains growths compared with that of W-AR34 due to the stronger DtsR1 expression (Figure 4). Other experiments on DtsR1 and AccBC combinations yielded similar results (Supplementary Figure S4). In conclusion, *C. glutamicum*–derived Acc can promote cell growth by regulating the expression levels of DtsR1 and AccBC, which may be beneficial for 3-HP production.

### The Effect of Regulated AccBC and DtsR1 on 3-HP Production

**Figure 5:**
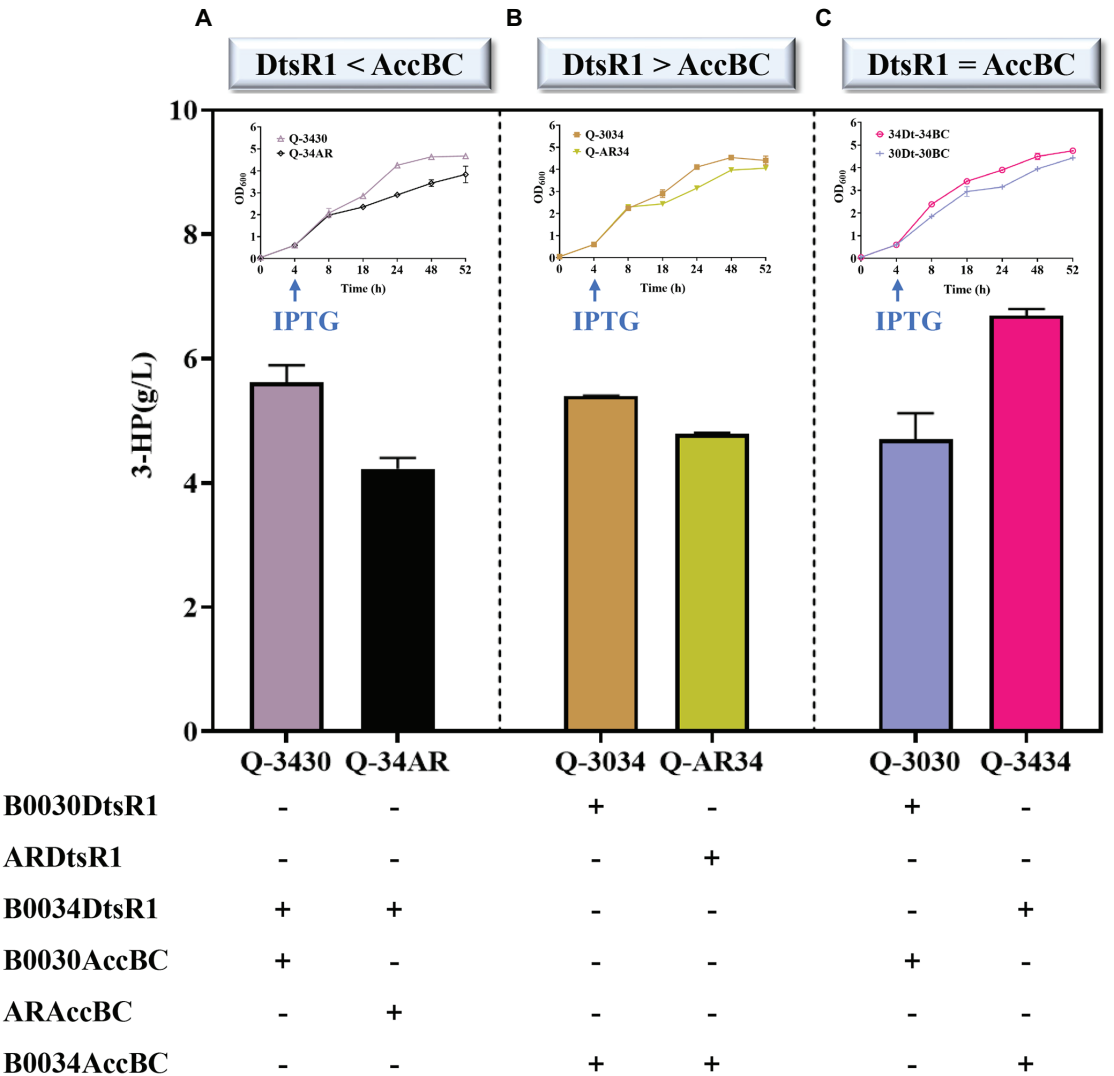
Biosynthesis of 3-HP with adjusted DtsR1 and AccBC expression in a producing strain Q2098. Three expression levels of DtsR1 and AccBC, namely strains **(A)** Q-3430/Q-34AR (DtsR1 < AccBC), **(B)** Q-3034/Q-AR34 (DtsR1 > AccBC), and **(C)** Q-3030/Q-3434 (DtsR1 = AccBC), were studied for their influence on 3-HP production. All results were calculated with three (*n* = 3) independent replicates.

To verify the influence of 3-HP synthesis through the modification of DtsR1 and AccBC expression levels, we constructed a series of strains containing DtsR1 and AccBC of various expression levels by fusing together different strengths of RBS in a producing strain Q2098 (Figure 5). Strain on a high level of AccBC (Q-3430) was beneficial for 3-HP accumulation and cell growth under the condition of AccBC expression stronger than DtsR1 (Figure 5A). Similarly, when the level of DtsR1 was higher than that of AccBC, 3-HP production and cell growth were simultaneously promoted in strain Q-3034 with a high level of DtsR1 (Figure 5B). When the expression level of DtsR1 and AccBC were the same, 3-HP production with Q-3434 (6.8 g/L) was higher than that with Q-3030 (4.7 g/L; Figure 5C). A study revealed that Acc activity is controlled through biotinylation by AccBC because it is a biotin-dependent carboxylase (Shi et al., 2014). DtsR1 can transfer carboxyl from BCCP-biotin into acetyl-CoA to form malonyl-CoA. Intracellular accumulation of acetyl-CoA and malonyl-CoA is tightly related to cell growth due to the key nodes through the connection with many pathways to regulate cell survival and biochemical synthesis (Chang et al., 1999; Zha et al., 2009; Krivoruchko et al., 2015; Shi and Tu, 2015). Therefore, the appropriate adjustment of catalyzing acetyl-CoA to malonyl-CoA can promoted 3-HP production and cell growth through the modulation between levels of DtsR1 and AccBC. These results indicated that 3-HP production is closely related to the modification of DtsR1 and AccBC expression levels. Robust cell host result in the improved 3-HP titer. Previous studies have successfully increased the production of malonyl-CoA-derived chemical compounds by using Acc overexpression (Miyahisa et al., 2005; Leonard et al., 2007; Wattanachaisaereekul et al., 2008). In this study, a high-efficiency strain Q-3434 was successfully constructed by adjusting the expression of DtsR1 and AccBC, leading to the production of 6.8 g/L of 3-HP. Therefore, 3-HP production through the malonyl-CoA pathway can be increased by adjusting the expression of DtsR1 and AccBC.

### Achieving a Balance Between Acc and MCR to Improve 3-HP Production

**Figure 6:**
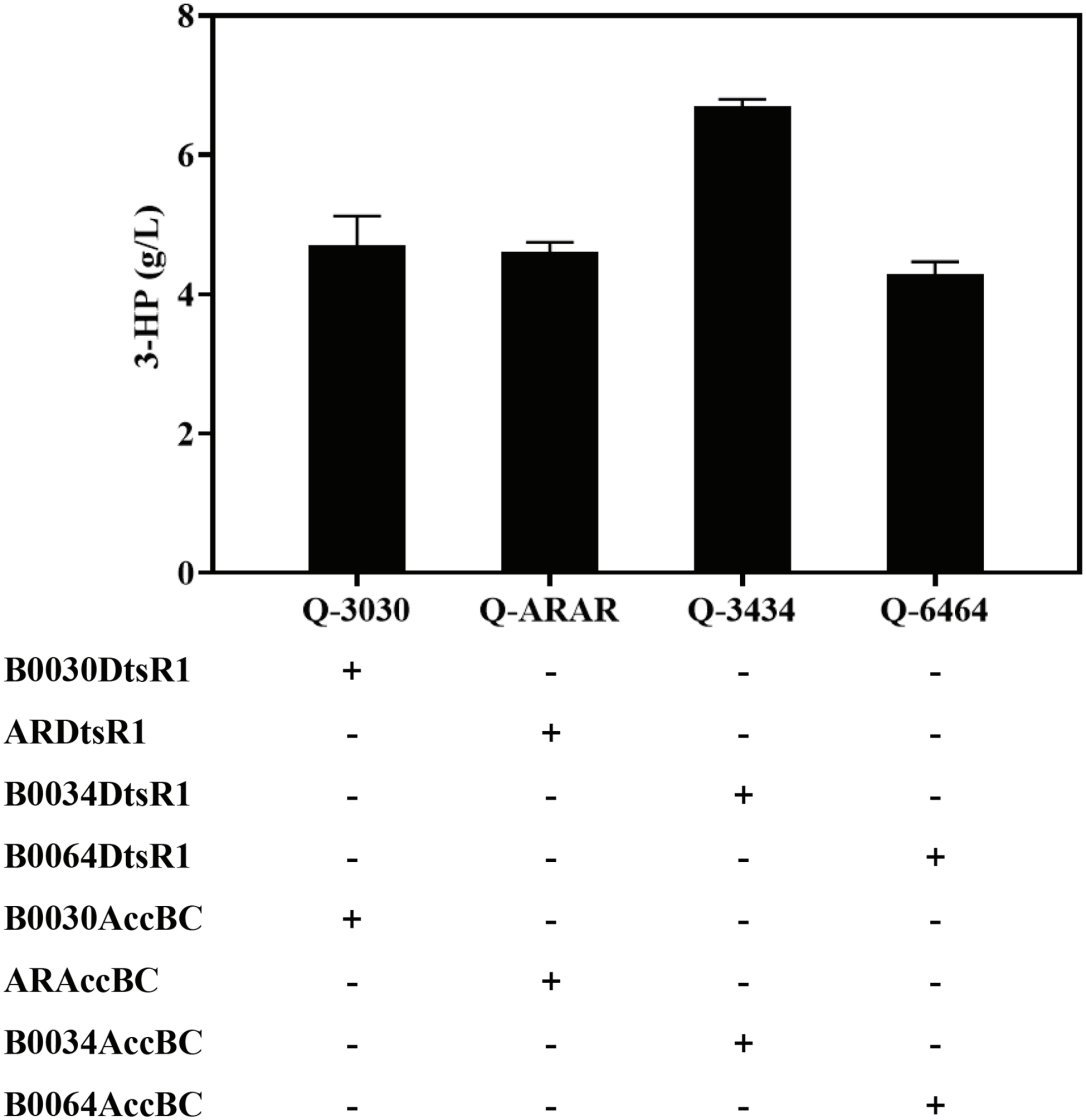
Balance of Acc and MCR expression levels for improving 3-HP production in a producing strain Q2098. Four levels of Acc (strains Q-3030, Q-ARAR, Q-3434, and Q-6464) were controlled by substituting RBSs of various strengths. 3-HP was detected in 48 h. All results were calculated with three (*n* = 3) independent replicates.

The catalysis efficiency of MCR improves through the attainment of a balance between the two separated fragments of MCR including mutated MCR-C and MCR-N, which reached a concentration of 3.72 g/L during shake-flask fermentation (Liu et al., 2016). However, the balance of Acc and MCR for 3-HP production had not been studied. In this study, the relationship of expressed Acc and MCR was investigated by fusing four strengths of RBS (high-B0030, medium-AR, weak-B0034, and weaker-B0064) with Acc expressing. Mutated MCR-C was expressed in another plasmid. These two plasmids were co-transformed into strain Q2098, an *E. coli* BL21 (DE3) integrated MCR-N, for 3-HP production. Compared with the high, medium and weaker expression levels of Acc, the weak level of Acc in strain Q-3434 achieved the highest titer of 6.8 g/L, yield of 0.566 g/g glucose, and productivity of 0.13 g/L/h during shake-flask fermentation through the malonyl-CoA pathway (Figure 6; Table 2). Additionally, Q-3434 produced less acetate and more lactate, which may be attributable to the efficient catalysis of acetyl-CoA to Malonyl-CoA with Acc as well as the increased glycolytic pathway (Supplementary Figure S5). Therefore, achieving a balance between Acc and MCR expression levels is vital for enhancing 3-HP production.

**Table 2:** Comparison of 3-HP production with glucose as the sole carbon source in *Escherichia coli*.

Production host	Strategies	Productivity (g/L/h)	Yield (g/g)	Titer (g/L)	Reactor	References
Q-3434	Regulating DtsR1, AccBC expression level and balancing strength of Acc and MCR	0.13 1.03	0.566 0.246	6.8 38.13	Shake flask 7.5 L bioreactor	This study
BE-MDA	Heterologous expressing acetyl-CoA carboxylase from *C. glutamicum*	0.04 0.40	0.18 –	1.80 10.08	Shake flask 5 L bioreactor	Cheng et al., 2016
Q2186	Balancing activity level of MCR-C and MCR-N	0.08 0.56	– 0.19	3.72 40.60	Shake flask 5 L bioreactor	Liu et al., 2016
Ec-MAP	Enhancing mal-CoA and NADPH supply	8.03E-3	–	0.19	Shake flask	Rathnasingh et al., 2012
pMCR-N-C	Increasing malonyl-CoA reductase activity by dissection	3.13E-3	–	0.15	Shake flask	Liu et al., 2013
CWF4NAS containing pTac15kPTA and p100-99A-DT12P	Introducing heterologous genes, pyruvate transaminase (encoded by *pa0132*), and overexpressing malonic acid reductase (encoded by *ydf G*), semialdehyde dehydrogenase (encoded by *yneI*) in *β*-alanine pathway	0.63	0.423	31.1	6.6 L bioreactor	Qin et al., 2020

### Fed-Batch Fermentation

**Figure 7:**
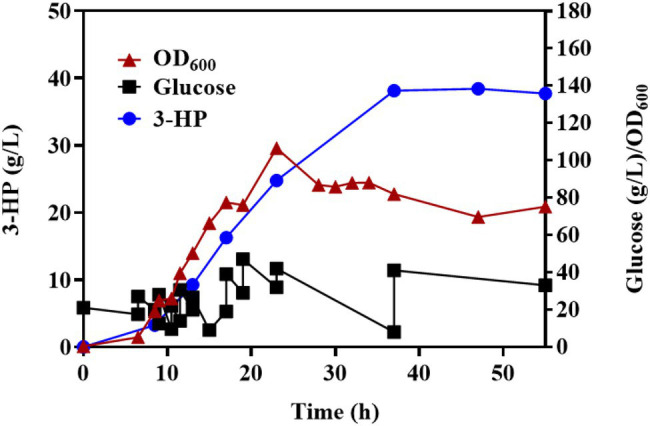
The fed-batch process with Q-3434 in a 7.5-L bioreactor. Biomass, glucose consumption, and 3-HP accumulation were monitored in real time.

To further verify the properties of Q-3434 for 3-HP production, we amplified the fermentation scale through fed-batch fermentation in a 7.5-L bioreactor. The cell density, 3-HP titer, glucose consumption levels, and by-products (acetate and lactate) are depicted (Figure 7). Following 37 h of cultivation, 38.13 g/L 3-HP was obtained, with a productivity and yield of 1.03 g/L/h and 0.246 g/g glucose concentration, respectively. After 37 h of incubation, unfavorable growth conditions and irreparable cellular damage lead to a death phase and long-term stationary phase (Finkel, 2006), resulting in the lose viability of 3-HP production and glucose consumption. The maximization of productivity, yield, and titer is crucial for achieving large-scale industrial production with high cost efficiency (Wendisch, 2020). Although studies had achieved a high titer (40.6 g/L of 3-HP) through the malonyl-CoA pathway, the actual yield (0.19 g/g glucose) has been much lower than the theoretical yield (1 g/g glucose), and the productivity (0.56 g/L/h) has been lower than that of other biochemicals (Li et al., 2013; Liu et al., 2016, 2019a; Meadows et al., 2016; Félix et al., 2019; Hao et al., 2020). In contrast to the results of other reports, Q-3434 achieved the highest productivity under a high yield and tier with cheap glucose as the sole carbon source (Table 2). Therefore, with respect to the three major parameters in industrial production, Q-3434 possesses substantial 3-HP production ability.

## Conclusion

Acc is the rate-limiting step for malonyl-CoA-derived metabolite production. However, the toxicity of overexpressed Acc for cell growth is the bottleneck to constructing an efficient microbial cell factory. Here, for the first time, we demonstrated that cell growth can be accelerated through the adjustment of the expression of two subunits (DtsR1 and AccBC) of *C. glutamicum*–derived Acc, which can be used to increase 3-HP production. Finally, Q-3434 was constructed through balancing Acc and MCR expression levels. In comparison with current reports, Q-3434 achieved a highest titer of 6.8 g/L and productivity of 1.03 g/L/h of 3-HP during shake-flask and fed-batch fermentation, respectively, with cheap glucose as the sole carbon source. Moreover, since Acc is the rate-limiting step for accumulating intracellular malonyl-CoA concentrations, this strategy might can be used to increase the chemical compound production of other malonyl-CoA-derivatives.

## Data Availability Statement

The original contributions presented in the study are included in the article/Supplementary Material, further inquiries can be directed to the corresponding authors.

## Author Contributions

SW: investigation, conceptualization, and writing—original draft. XJ: data curation and formal analysis. WJ: data curation and methodology. QW: methodology. QQ and QL: conceptualization, funding acquisition, resources, project administration, supervision, and writing—review and editing. All authors contributed to the article and approved the submitted version.

## Funding

This work has been supported by the National Key Research and Development Program of China (2019YFA0706900) and National Natural Science Foundation of China (31971336 and 31770095).

## Conflict of Interest

The authors declare that the research was conducted in the absence of any commercial or financial relationships that could be construed as a potential conflict of interest.

## Publisher’s Note

All claims expressed in this article are solely those of the authors and do not necessarily represent those of their affiliated organizations, or those of the publisher, the editors and the reviewers. Any product that may be evaluated in this article, or claim that may be made by its manufacturer, is not guaranteed or endorsed by the publisher.

## Footnotes

^1^ http://parts.igem.org/Main_Page

## Abbreviations

Acc: Acetyl-CoA carboxylase
BC: Biotin carboxylase
HPLC: High-performance liquid chromatography
3-HP: 3-hydroxypropionic acid
IPTG: Isopropyl-β-d-thiogalactoside
MCR: Malonyl-CoA reductase
NADPH: Nicotinamide adenine dinucleotide phosphate
RBS: Ribosome bind site
