# Supplementary material for: The Expression Modulation of the Key Enzyme Acc for Highly Efficient 3-Hydroxypropionic Acid Production
Source: Front Microbiol. 2022 May 11;13:902848. doi: 10.3389/fmicb.2022.902848 (PMC9130761; doi:10.3389/fmicb.2022.902848)
Supplement: Supplementary file 1 [file Data_Sheet_1.docx]

**Supplementary materials**

**Supplementary Table S1.** Plasmids used in this study

| Plasmids | Description | Source |
| --- | --- | --- |
| pACYCDuet-1 | rep_p15A_ Cm^R^ *lacI* P_T7_ | Novagen |
| pETDuet-1 | rep_pBR322_ Amp^R^ *lacI* P_T7_ | Novagen |
| pMCR-C-N940V/K1106W/S1114R | repp_BR322_ Amp^R^ lacI P_T7_ *His6-mcr550-1219* N940V K1106W S1 1 14R | (Liu et al., 2016) |
| pA-DtsR1-AccBC | rep_p15A_ Cm^R^ *lacI* P_T7_ *DtsR1* P_T7_ *accBC* from *C. glutamicum* | This study |
| pA-DtsR1 | rep_p15A_ Cm^R^ *lacI* P_T7_ d*tsR1* from *C. glutamicum* | This study |
| pA-AccBC | rep_p15A_ Cm^R^ *lacI* P_T7_ *accBC* from *C. glutamicum* | This study |
| pA-0029 | rep_p15A_ Cm^R^ *lacI* P_T7_ B0029-RFP | This study |
| pA-0030 | rep_p15A_ Cm^R^ *lacI* P_T7_ B0030-RFP | This study |
| pA-0031 | rep_p15A_ Cm^R^ *lacI* P_T7_ B0031-RFP | This study |
| pA-0032 | rep_p15A_ Cm^R^ *lacI* P_T7_ B0032-RFP | This study |
| pA-0033 | rep_p15A_ Cm^R^ *lacI* P_T7_ B0033-RFP | This study |
| pA-0034 | rep_p15A_ Cm^R^ *lacI* P_T7_ B0034-RFP | This study |
| pA-0035 | rep_p15A_ Cm^R^ *lacI* P_T7_ B0035-RFP | This study |
| pA-0064 | rep_p15A_ Cm^R^ *lacI* P_T7_ B0064-RFP | This study |
| pA-AR | rep_p15A_ Cm^R^ *lacI* P_T7_ AR-RFP | This study |
| D30-AAR | rep_p15A_ Cm^R^ *lacI* P_T7_ B0030-d*tsR1*-AR-*accBC* from *C. glutamicum* | This study |
| D30-A34 | rep_p15A_ Cm^R^ *lacI* P_T7_ B0030-*dtsR1*-B0034-*accBC* from *C. glutamicum* | This study |
| DAR-A34 | rep_p15A_ Cm^R^ *lacI* P_T7_ AR-*dtsR1*-B0034-*accBC* from *C. glutamicum* | This study |
| DAR-A30 | rep_p15A_ Cm^R^ *lacI* P_T7_ AR-*dtsR1*-B0030-*accBC* from *C. glutamicum* | This study |
| D34-A30 | rep_p15A_ Cm^R^ *lacI* P_T7_ B0034-*dtsR1*-B0030-*accBC* from *C. glutamicum* | This study |
| D34-AAR | rep_p15A_ Cm^R^ *lacI* P_T7_ B0034-*dtsR1*-AR-*accBC* from *C. glutamicum* | This study |
| D30-A30 | rep_p15A_ Cm^R^ *lacI* P_T7_ B0030-*dtsR1*-B0030-*accBC* from *C. glutamicum* | This study |
| DAR-AAR | rep_p15A_ Cm^R^ *lacI* P_T7_ AR-*dtsR1*-AR-*accBC* from *C. glutamicum* | This study |
| D34-A34 | rep_p15A_ Cm^R^ *lacI* P_T7_ B0034-*dtsR1*-B0034-*accBC* from *C. glutamicum* | This study |
| D64-A64 | rep_p15A_ Cm^R^ *lacI* P_T7_ B0064-*dtsR1*-B0064-*accBC* from *C. glutamicum* | This study |

**Supplementary Table S2.** Primers used in this study

| Primer name | Sequences (5’-3’) |
| --- | --- |
| pA-DtsR1-AccBC reconstruction |  |
| pA-F1 | GCTCGCAAGCACGGCAACATGCCACTGTAAAATTCGAGCTCGGCGCGCCT |
| pA-R1 | GACGTCAATCAAAGGTGAGGAAATGGTCATCGGATCCTGGCTGTGGTGATGAT |
| pA-F2 | AAGGGCGTTGTTCTCCTCGAGATCAAGTAACTCGAGTCTGGTAAAGAAACCG |
| pA-R2 | CTTGGTGATCTTCCTAGTCTCGACTGACACCATATGTATATCTCCTTCTTATAC |
| pA-DtsR1-F | ATGACCATTTCCTCACCTTTG |
| pA-DtsR1-R | TTACAGTGGCATGTTGCCGTG |
| pA-accBC-F | GTGTCAGTCGAGACTAGGAAG |
| pA-accBC-R | TTACTTGATCTCGAGGAGAAC |
| pA-DtsR1 reconstruction |  |
| pA-DtsR1-F | ATGACCATTTCCTCACCTTTG |
| pA-DtsR1-R | TTACAGTGGCATGTTGCCGTG |
| pA-F3 | CACGGCAACATGCCACTGTAACTCGAGTCTGGTAAAGAAACCGCTGCTGCG |
| pA-R1 | GACGTCAATCAAAGGTGAGGAAATGGTCATCGGATCCTGGCTGTGGTGATGAT |
| pA-AccBC reconstruction |  |
| pA-accBC-F2 | TCTAGAGATTAAAGAGGAGAAATACTAGGTGTCAGTCGAGACTAGGAAG |
| pA-accBC-R | TTACTTGATCTCGAGGAGAAC |
| pA-F2 | AAGGGCGTTGTTCTCCTCGAGATCAAGTAACTCGAGTCTGGTAAAGAAACCG |
| pA-R3 | ACCTAGTATTTCTCCTCTTTAATCTCTAGACTACAGGGGAATTGTTATCCGCT |
| pA-0029 reconstruction |  |
| RFP-F | ATGGCTTCCTCCGAAGACGTTAT |
| RFP-R | TTAAGCGTAGTTTTCGTCGTTTGC |
| pA-0029-F | GCTGCAGCAAACGACGAAAACTACGCTTAACTCGAGTCTGGTAAAGAAACCG |
| pA-0029-R | CTTTGATAACGTCTTCGGAGGAAGCCATCTAGTAGGTTTCCTGTGTGAACTCTAGACTACAGGGGAATTGTTATCCGC |
| pA-0030 reconstruction |  |
| RFP-F | ATGGCTTCCTCCGAAGACGTTAT |
| RFP-R | TTAAGCGTAGTTTTCGTCGTTTGC |
| pA-0029-F | GCTGCAGCAAACGACGAAAACTACGCTTAACTCGAGTCTGGTAAAGAAACCG |
| pA-0030-R | CTTTGATAACGTCTTCGGAGGAAGCCATCTAGTATTTCTCCTCTTTAATCTCTAGACTACAGGGGAATTGTTATCCGC |
| pA-0031 reconstruction |  |
| RFP-F | ATGGCTTCCTCCGAAGACGTTAT |
| RFP-R | TTAAGCGTAGTTTTCGTCGTTTGC |
| pA-0029-F | GCTGCAGCAAACGACGAAAACTACGCTTAACTCGAGTCTGGTAAAGAAACCG |
| pA-0031-R | TCTTTGATAACGTCTTCGGAGGAAGCCATCTAGTAGGTTTCCTGTGTGACTCTAGACTACAGGGGAATTGTTATCCGC |
| pA-0032 reconstruction |  |
| RFP-F | ATGGCTTCCTCCGAAGACGTTAT |
| RFP-R | TTAAGCGTAGTTTTCGTCGTTTGC |
| pA-0029-F | GCTGCAGCAAACGACGAAAACTACGCTTAACTCGAGTCTGGTAAAGAAACCG |
| pA-0032-R | CTCTTTGATAACGTCTTCGGAGGAAGCCATCTAGTACTTTCCTGTGTGACTCTAGACTACAGGGGAATTGTTATCCGC |
| pA-0033 reconstruction |  |
| RFP-F | ATGGCTTCCTCCGAAGACGTTAT |
| RFP-R | TTAAGCGTAGTTTTCGTCGTTTGC |
| pA-0029-F | GCTGCAGCAAACGACGAAAACTACGCTTAACTCGAGTCTGGTAAAGAAACCG |
| pA-0033-R |  |
| pA-0034 reconstruction |  |
| RFP-F | ATGGCTTCCTCCGAAGACGTTAT |
| RFP-R | TTAAGCGTAGTTTTCGTCGTTTGC |
| pA-0029-F | GCTGCAGCAAACGACGAAAACTACGCTTAACTCGAGTCTGGTAAAGAAACCG |
| pA-0034-R | ACTCTTTGATAACGTCTTCGGAGGAAGCCATCTAGTATTTCTCCTCTTTCTCTAGACTACAGGGGAATTGTTATCCGC |
| pA-0035 reconstruction |  |
| RFP-F | ATGGCTTCCTCCGAAGACGTTAT |
| RFP-R | TTAAGCGTAGTTTTCGTCGTTTGC |
| pA-0029-F | GCTGCAGCAAACGACGAAAACTACGCTTAACTCGAGTCTGGTAAAGAAACCG |
| pA-0035-R | TCTTTGATAACGTCTTCGGAGGAAGCCATCTAGTATTCTCCTCTTTAATCTCTAGACTACAGGGGAATTGTTATCCGC |
| pA-0064 reconstruction |  |
| RFP-F | ATGGCTTCCTCCGAAGACGTTAT |
| RFP-R | TTAAGCGTAGTTTTCGTCGTTTGC |
| pA-0029-F | GCTGCAGCAAACGACGAAAACTACGCTTAACTCGAGTCTGGTAAAGAAACCG |
| pA-0064-R | ACTCTTTGATAACGTCTTCGGAGGAAGCCATCTAGTATTTCCCCTCTTTCTCTAGACTACAGGGGAATTGTTATCCGC |
| pA-AR reconstruction |  |
| RFP-F | ATGGCTTCCTCCGAAGACGTTAT |
| RFP-R | TTAAGCGTAGTTTTCGTCGTTTGC |
| pA-0029-F | GCTGCAGCAAACGACGAAAACTACGCTTAACTCGAGTCTGGTAAAGAAACCG |
| pA-RBS-R | CTCTTTGATAACGTCTTCGGAGGAAGCCATGCTGCTGCCCATGGTATATCT |
| D30-AAR reconstruction |  |
| D1-F | AGTCTAGAGATTAAAGAGGAGAAATACTAGATGACCATTTCCTCACCTTTG |
| D1-R | ATCTCCTTATTAAAGTTAAACAAAATTATTTCTACAGGTTACAGTGGCATGTTGCCGTG |
| A1-F | GTTTAACTTTAATAAGGAGATATACCATGGGCAGCAGCGTGTCAGTCGAGACTAGGAAG |
| pA-accBC-R | TTACTTGATCTCGAGGAGAAC |
| pA-F2 | AAGGGCGTTGTTCTCCTCGAGATCAAGTAACTCGAGTCTGGTAAAGAAACCG |
| BB1-R | ATCTAGTATTTCTCCTCTTTAATCTCTAGACTACAGGGGAATTGTTATCCGC |
| D30-A34 reconstruction |  |
| D1-F | AGTCTAGAGATTAAAGAGGAGAAATACTAGATGACCATTTCCTCACCTTTG |
| D2-R | GACACCTAGTATTTCTCCTCTTTCTCTAGATTACAGTGGCATGTTGCCGTG |
| A2-F | TGTAATCTAGAGAAAGAGGAGAAATACTAGGTGTCAGTCGAGACTAGGAAG |
| pA-accBC-R | TTACTTGATCTCGAGGAGAAC |
| pA-F2 | AAGGGCGTTGTTCTCCTCGAGATCAAGTAACTCGAGTCTGGTAAAGAAACCG |
| BB1-R | ATCTAGTATTTCTCCTCTTTAATCTCTAGACTACAGGGGAATTGTTATCCGC |
| DAR-A34 reconstruction |  |
| pA-DtsR1-F | ATGACCATTTCCTCACCTTTG |
| D2-R | GACACCTAGTATTTCTCCTCTTTCTCTAGATTACAGTGGCATGTTGCCGTG |
| A2-F | TGTAATCTAGAGAAAGAGGAGAAATACTAGGTGTCAGTCGAGACTAGGAAG |
| pA-accBC-R | TTACTTGATCTCGAGGAGAAC |
| pA-F2 | AAGGGCGTTGTTCTCCTCGAGATCAAGTAACTCGAGTCTGGTAAAGAAACCG |
| BB4-R | GACGTCAATCAAAGGTGAGGAAATGGTCATGCTGCTGCCCATGGTATATCT |
| DAR-A30 reconstruction |  |
| pA-DtsR1-F | ATGACCATTTCCTCACCTTTG |
| DT-R | ACCTAGTATTTCTCCTCTTTAATCTCTAGATTACAGTGGCATGTTGCCGTG |
| AC-F | TCTAGAGATTAAAGAGGAGAAATACTAGGTGTCAGTCGAGACTAGGAAG |
| pA-accBC-R | TTACTTGATCTCGAGGAGAAC |
| pA-F2 | AAGGGCGTTGTTCTCCTCGAGATCAAGTAACTCGAGTCTGGTAAAGAAACCG |
| BB4-R | GACGTCAATCAAAGGTGAGGAAATGGTCATGCTGCTGCCCATGGTATATCT |
| D34-A30 reconstruction |  |
| D6-F | TGTAGTCTAGAGAAAGAGGAGAAATACTAGATGACCATTTCCTCACCTTTG |
| DT-R | ACCTAGTATTTCTCCTCTTTAATCTCTAGATTACAGTGGCATGTTGCCGTG |
| AC-F | TCTAGAGATTAAAGAGGAGAAATACTAGGTGTCAGTCGAGACTAGGAAG |
| pA-accBC-R | TTACTTGATCTCGAGGAGAAC |
| pA-F2 | AAGGGCGTTGTTCTCCTCGAGATCAAGTAACTCGAGTCTGGTAAAGAAACCG |
| BB6-R | GTCATCTAGTATTTCTCCTCTTTCTCTAGACTACAGGGGAATTGTTATCCGC |
| D34-AAR reconstruction |  |
| D6-F | TGTAGTCTAGAGAAAGAGGAGAAATACTAGATGACCATTTCCTCACCTTTG |
| D1-R | ATCTCCTTATTAAAGTTAAACAAAATTATTTCTACAGGTTACAGTGGCATGTTGCCGTG |
| A1-F | GTTTAACTTTAATAAGGAGATATACCATGGGCAGCAGCGTGTCAGTCGAGACTAGGAAG |
| pA-accBC-R | TTACTTGATCTCGAGGAGAAC |
| pA-F2 | AAGGGCGTTGTTCTCCTCGAGATCAAGTAACTCGAGTCTGGTAAAGAAACCG |
| BB6-R | GTCATCTAGTATTTCTCCTCTTTCTCTAGACTACAGGGGAATTGTTATCCGC |
| D30-A30 reconstruction |  |
| D1-F | AGTCTAGAGATTAAAGAGGAGAAATACTAGATGACCATTTCCTCACCTTTG |
| DT-R | ACCTAGTATTTCTCCTCTTTAATCTCTAGATTACAGTGGCATGTTGCCGTG |
| AC-F | TCTAGAGATTAAAGAGGAGAAATACTAGGTGTCAGTCGAGACTAGGAAG |
| pA-accBC-R | TTACTTGATCTCGAGGAGAAC |
| pA-F2 | AAGGGCGTTGTTCTCCTCGAGATCAAGTAACTCGAGTCTGGTAAAGAAACCG |
| BB1-R | ATCTAGTATTTCTCCTCTTTAATCTCTAGACTACAGGGGAATTGTTATCCGC |
| DAR-AAR reconstruction |  |
| pA-DtsR1-F | ATGACCATTTCCTCACCTTTG |
| D1-R | ATCTCCTTATTAAAGTTAAACAAAATTATTTCTACAGGTTACAGTGGCATGTTGCCGTG |
| A1-F | GTTTAACTTTAATAAGGAGATATACCATGGGCAGCAGCGTGTCAGTCGAGACTAGGAAG |
| pA-accBC-R | TTACTTGATCTCGAGGAGAAC |
| pA-F2 | AAGGGCGTTGTTCTCCTCGAGATCAAGTAACTCGAGTCTGGTAAAGAAACCG |
| BB4-R | GACGTCAATCAAAGGTGAGGAAATGGTCATGCTGCTGCCCATGGTATATCT |
| D34-A34 reconstruction |  |
| D6-F | TGTAGTCTAGAGAAAGAGGAGAAATACTAGATGACCATTTCCTCACCTTTG |
| D2-R | GACACCTAGTATTTCTCCTCTTTCTCTAGATTACAGTGGCATGTTGCCGTG |
| A2-F | TGTAATCTAGAGAAAGAGGAGAAATACTAGGTGTCAGTCGAGACTAGGAAG |
| pA-accBC-R | TTACTTGATCTCGAGGAGAAC |
| pA-F2 | AAGGGCGTTGTTCTCCTCGAGATCAAGTAACTCGAGTCTGGTAAAGAAACCG |
| BB6-R | GTCATCTAGTATTTCTCCTCTTTCTCTAGACTACAGGGGAATTGTTATCCGC |
| D64-A64 reconstruction |  |
| D9-F | TGTAGTCTAGAGAAAGAGGGGAAATACTAGATGACCATTTCCTCACCTTTG |
| D3-R | GACACCTAGTATTTCCCCTCTTTCTCTAGATTACAGTGGCATGTTGCCGTG |
| A3-F | TGTAATCTAGAGAAAGAGGGGAAATACTAGGTGTCAGTCGAGACTAGGAAG |
| pA-accBC-R | TTACTTGATCTCGAGGAGAAC |
| pA-F2 | AAGGGCGTTGTTCTCCTCGAGATCAAGTAACTCGAGTCTGGTAAAGAAACCG |
| BB9-R | GTCATCTAGTATTTCCCCTCTTTCTCTAGACTACAGGGGAATTGTTATCCGC |

**Supplementary Table S3.** Characterized RBS sequences in this study

| RBS Name | Sequences |
| --- | --- |
| B0029 | TCTAGAGTTCACACAGGAAACCTACTAG |
| B0030 | TCTAGAGATTAAAGAGGAGAAATACTAG |
| B0031 | TCTAGAGTCACACAGGAAACCTACTAG |
| B0032 | TCTAGAGTCACACAGGAAAGTACTAG |
| B0033 | TCTAGAGTCACACAGGACTACTAG |
| B0034 | TCTAGAGAAAGAGGAGAAATACTAG |
| B0035 | TCTAGAGATTAAAGAGGAGAATACTAG |
| B0064 | TCTAGAGAAAGAGGGGAAATACTAG |
| AR | AAATAATTTTGTTTAACTTTAATAAGGAGATATACCATGGGCAGCAGC |


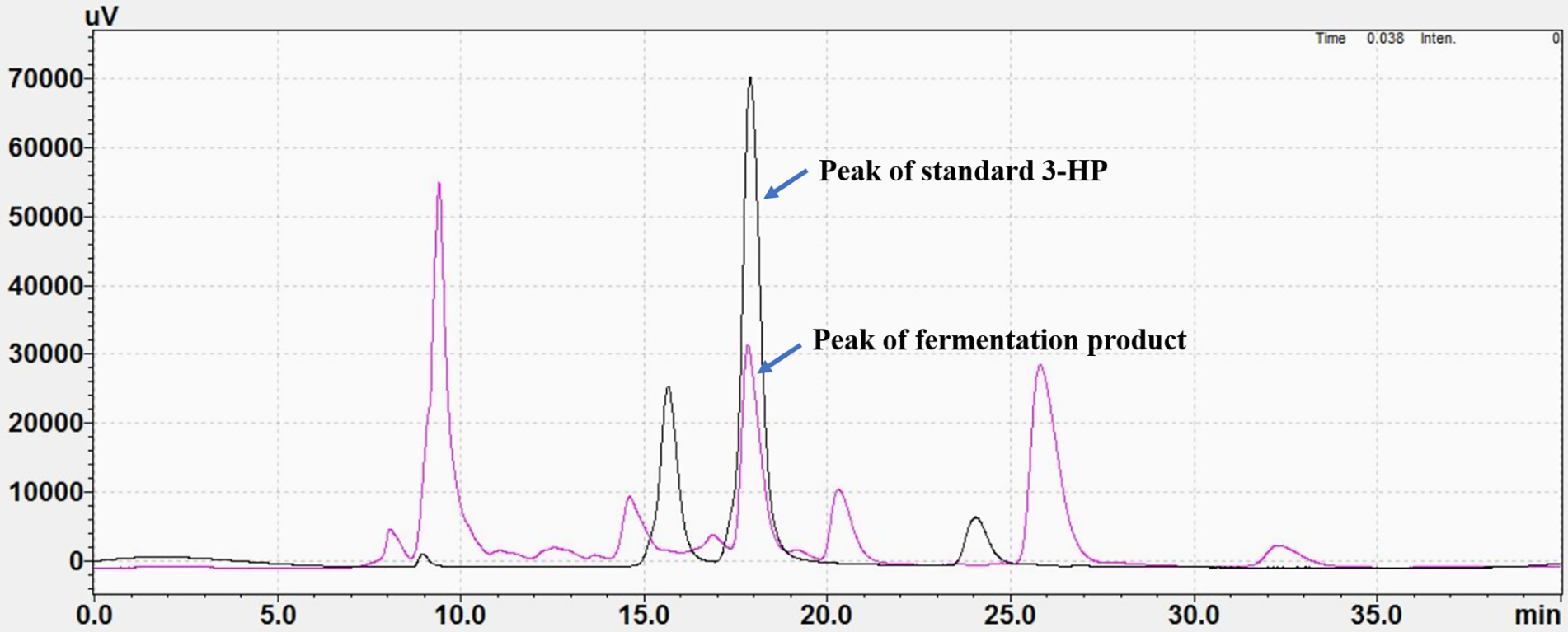


**Supplementary Figure S1.** Comparation of 3-HP peaks in standard 3-HP and the fermentation product with HPLC detection method.


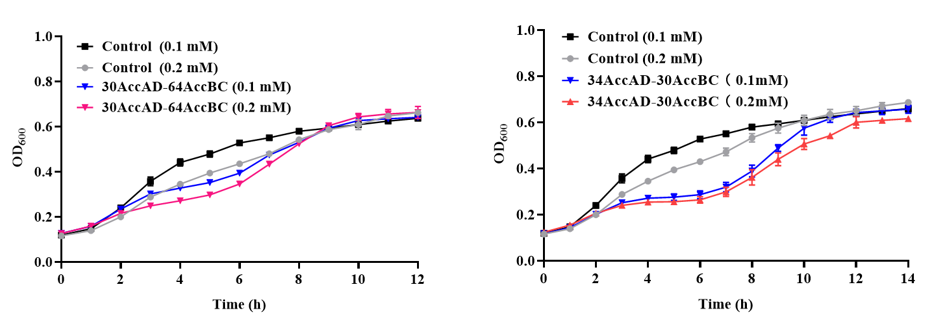
**
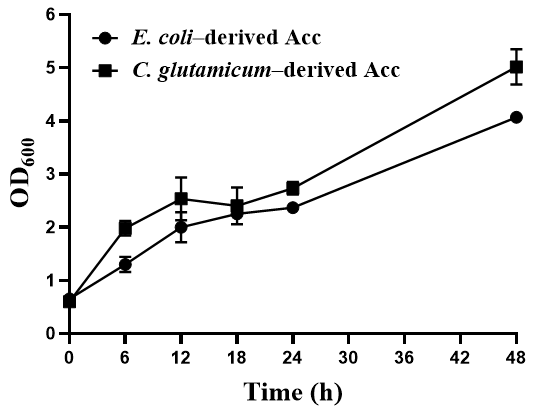
Supplementary Figure S2.** Comparation of cell growth in *E. coli* BL21 (DE3) between engineered strains *E. coli-derived* Acc and *E. coli-derived* Acc. All results were calculated with three (n=3) independent replicates.

**Supplementary Figure S3.** Repression of overexpressed native Acc for cell growth in *E. coli* BL21 (DE3). Strain Control containing empty vector as control. Strain 30AccAD-64AccBC containing RBS B0030 and B0064 for *accAD* and *accBC* respectively, which indicated that *accAD* expressed higher than *accBC*. Strain 34AccAD-30AccBC containing RBS B0034 and B0030 for *accAD* and *accBC* respectively, which indicated that *accAD* expressed lower than *accBC*. All strains were induced by 0.1 mM or 0.2 mM IPTG. All results were calculated with three (n=3) independent replicates.


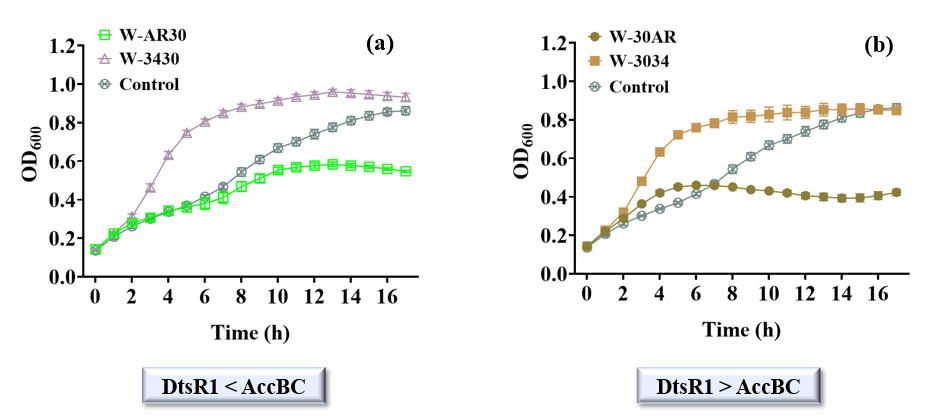


**Supplementary Figure S4.** Analysis of adjusted DtsR1 and AccBC for their influence on cell growth in *E. coli* BL21 (DE3). (a) DtsR1 expressed lower than AccBC: Strain ARDtsR1-30AccBC containing RBS AR and B0030 for DtsR1 and AccBC respectively. Strain 34DtsR1-30AccBC containing RBS B0034 and B0030 for DtsR1 and AccBC respectively. (b) DtsR1 expressed higher than AccBC: Strain 30DtsR1-ARAccBC containing RBS B0030 and AR for DtsR1 and AccBC respectively. Strain 30DtsR1-34AccBC containing RBS B0030 and B0034 for DtsR1 and AccBC respectively. All strains were induced by 0.1 mM or 0.2 mM IPTG. All results were calculated with three (n=3) independent replicates.


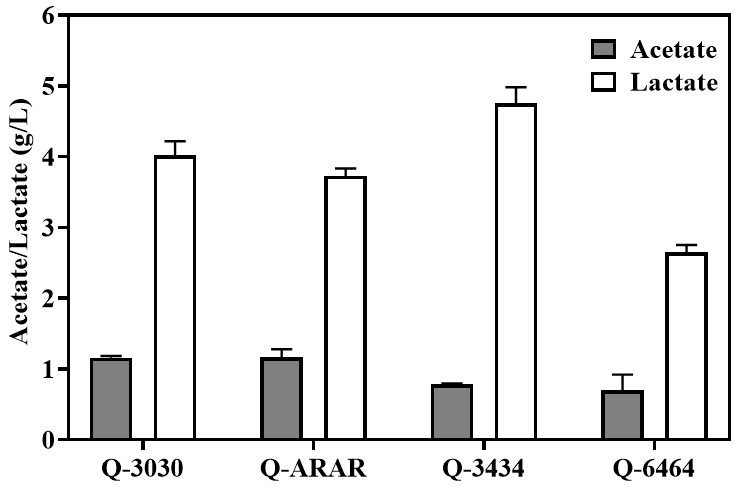
**Supplementary Figure S5.** Detection of acetate and lactate in 3-HP producing strains with different Acc levels. Four levels of Acc (strains Q-3030, Q-ARAR, Q-3434, and Q-6464) were controlled by substituting RBSs of various strengths. Acetate and lactate were detected in 48 h. All results were calculated with three (n=3) independent replicates.
